# Early childhood caries, primary caregiver oral health knowledge and behaviours and associated sociological factors in Australia: a systematic scoping review

**DOI:** 10.1186/s12903-021-01887-4

**Published:** 2021-10-13

**Authors:** Lesley Andrew, Ruth Wallace, Nicole Wickens, Jilen Patel

**Affiliations:** 1 School of Medical and Health Sciences, Edith Cowan University, Joondalup, Australia; 2 Dental School, University of Western Australia, Perth, Australia

**Keywords:** Early childhood caries, Western Australia, Australia, Systematic scoping review, Primary caregiver knowledge and behaviours, Child dental benefit schedule

## Abstract

**Background:**

Early childhood caries disproportionately affects vulnerable groups and remains a leading cause of preventable hospital admissions for Western Australian children. The Western Australia State Oral Health Plan seeks to improve child oral health through universal and targeted health promotion initiatives with primary caregivers. These initiatives require evidence of primary caregiver oral health knowledge and behaviours and baseline data on early childhood caries. The objective of this systematic scoping review was to understand current oral health knowledge and practices of primary caregivers of children aged 0–4 years, identify influential socioecological determinants, and identify data on early childhood caries in the Western Australian context.

**Methods:**

A systematic scoping review framework identified articles published between 2010 and 2021, using Scopus, PubMed, Medline, CINAHL, PsycINFO, selected article reference lists, and oral health websites. The lack of Western Australian specific literature prompted the inclusion of Australia-wide articles. Articles were screened via author consensus, with eight selected.

**Results:**

Western Australia and nation-wide data on early childhood caries are limited and mostly dated. WA data from children aged 2–3 years, collected in 2006, suggests the prevalence is 2.9% in this state, with national data of children from 0 to 3 years, collected from 2006 and 2008, suggesting an early childhood caries prevalence of 3.4–8% of children aged 18 months, rising sharply by 36 months of age. Nationally, fewer than half the primary caregivers reported following evidence-based oral health recommendations for their young children. Perceptions of the role of dental services for young children tends to be focussed on treatment, rather than surveillance and prevention. Knowledge of dietary and oral hygiene practices is inconsistent and awareness of the Child Dental Benefit Schedule low. Young children’s oral health status is clearly associated with socioecological factors, including socioeconomic status.

**Conclusions:**

Recent early childhood caries data and evidence of primary care-givers’ oral health knowledge and behaviours are unavailable in Western Australia, a similar situation exists nationwide. To realise the Western Australian and National Oral Health Plans, research is required to address this knowledge gap.

## Background

### Child oral health in Australia

Dental caries, colloquially known as tooth decay, remains among the most common chronic conditions affecting children globally [1]. Dental caries is largely preventable; however, it disproportionately affects disadvantaged groups across all age groups ranging from infancy through to the elderly and the widening disparities in oral health has led to dental caries being labelled a ‘silent epidemic’ [2]. Early childhood caries (ECC) is a recognised public health concern and is defined as the presence of one or more decayed (non-cavitated or cavitated lesions), missing (due to caries) or filled tooth surfaces in any primary tooth in a child under 71 months of age [3]. ECC significantly impacts a child’s quality of life and untreated caries often results in pain, eating difficulties, development and sleep problems, time off school, and social embarrassment [4, 5]. The impact of ECC also affects the child’s family, with parents facing the financial impact of treatment fees and time off work to care for their unwell child [4]. Optimal oral health practices and early intervention have the potential to reduce the incidence of ECC and positively impact both oral and general health throughout the life-course with implications on the wellbeing of the individual and the community [6].

Dental caries is the most common chronic childhood condition in Australia, with almost half of all Australian pre-school children experiencing caries [7]. The prevalence of ECC is notably higher in children from low socio-economic, remote and Indigenous backgrounds [7]. In Western Australia (WA), an analysis of hospital data gathered between 2000 and 2009 found oral health problems, primarily caries, caused the highest rates of acute preventable hospitalisation admissions for children from 0 to 14 years of age [8]. Across these dates, 44,000 children were hospitalised, at an estimated cost to families and hospitals of over $92 million [8]. Emergency dental hospitalisation for relief of dental pain and infection remains a leading cause of potentially preventable hospitalisation in WA with the majority of preschool children requiring treatment under general anaesthesia [9]. Although associated mortality of general anaesthesia in young children is low, emergency hospitalisation is often emotionally distressing for the child and their parent and carries significant financial costs to public health infrastructure [10].

### Oral health promotion behaviours

Primary caregivers play a pivotal role in their child’s health and subsequent behaviours. There is a growing body of evidence that reflects the association between parental oral health knowledge and behaviours and their child’s oral health status [11]. Primary caregivers’ awareness and practice of oral health promoting diets, oral hygiene practices—including brushing teeth—and engagement with preventative dental services provide the foundation for their child’s continuing oral health throughout life [12].

A strong link has been established between ECC and frequent sugary food and drink consumption, nocturnal milk bottle use and on demand feeding [13, 14]. Furthermore, irregular brushing habits, not using age-appropriate fluoridated toothpaste and limited access to community dental care also significantly increase a child’s caries risk [15].

A dental home is defined by the American Academy of Pediatric Dentistry (AAPD) as *“*the ongoing relationship between the dentist and the patient, inclusive of all aspects of oral health care delivered in a comprehensive, continuously accessible, coordinated, and family-centered way” [16, p. 43]. Children with a dental home are more likely to receive appropriate preventive and routine oral health care and this has the potential to improve a family’s oral health knowledge and awareness around ECC [17]. The Australian guidelines, as well as the WA State Oral Health Plan, recommend that a child’s first dental visit should be scheduled once their first tooth erupts, or by the age of one year [18, 19].

In Australia, dental fees are not covered by Medicare (the publicly funded national universal health care service that offers subsidised, and in some situations, free health care), and dental health is identified separately from other categories of physical health. As such, financial barriers and cost of care have been negatively associated with dental attendance patterns while public dental pathways vary among individual states and territories [20]. In WA, the School Dental Service is available to children aged 5–16 years and provides routine dental care, including examinations, fillings, extractions, dental cleaning, radiographs and oral hygiene/tooth brushing instruction [21]. The service is staffed primarily by dental therapists who are supervised by dentists with more complex procedures requiring referral to specialist services [21]. However, unlike some other Australian states, such as Tasmania [22], children under five years of age in WA are not eligible to be seen through the School Dental Service. The Child Dental Benefit Schedule (CDBS) was introduced in 2014 as a supplemental avenue of care for children aged two years to 17 years for families that receive family tax benefits. The CDBS provides up to $1000 (every 2 years) for basic dental services such as dental examinations, cleaning, fillings, extractions, and X-rays [23]. However, only 10% of eligible WA families access the benefits of this scheme, compared to around 30% nationally. Moreover, very young children who present with extensive treatment needs requiring treatment under general anaesthesia are not covered by the scheme. While the reasons behind these differences are unclear, they demonstrate a clear inequality of opportunity for WA children.

### Primary caregiver oral health knowledge and behaviours

Improving primary caregivers’ understanding and practices around their children’s oral health is central to reducing existing oral health disparities and enabling sustainable outcomes [24]. Although scarce, a few Australian studies offer some evidence of primary caregiver oral health practices and knowledge of cariogenic food and drinks.

Conducted in 2018, a national study of 2000 households indicated low levels of a range of child oral health promoting knowledge as well as suboptimal oral health promoting practices among Australian primary caregivers. This study reported 31% of pre-schoolers had never been taken to a dentist, and 39–50% of children aged 0–3 years did not have their teeth cleaned at least twice a day [25]. This is supported by the results of the National Oral Health Study where 1 in 5 children aged 2–14 years had never consulted a dentist or dental professional [7]. Around 23% of primary caregiver survey participants indicated a belief that dental services were only accessible for treatment rather than prevention purposes and 77% were not aware their child should be taken to a dentist for their first visit by the age of 12 months [25]. Only 50% of participants were aware that fluoridated tap water was better for their children’s teeth than bottled water; a similar percentage were unaware of the CDBS. Moreover, around 85% did not know the recommended maximum daily intake of free sugars in their children’s diet [25].

A further study of over 2000 children and primary caregivers in Adelaide, South Australia reported the intake of free sugars among 73% of participants aged 0–2 years exceeded WHO recommendations [26]. This concerning evidence supports the need for specific research into WA primary caregivers’ oral health knowledge and practices with their children.

### The Australian National Oral Health Plan and the WA State Oral Health Plan

The Australian Government recognises the burden of poor oral health among the Australian population and the disparity in prevention and treatment accessibility for those on low income. The National Oral Health Plan 2015–2024 [27] outlines national goals to address these issues through: (1) health promotion (evidence-based activities that addresses the social determinants affecting oral health), (2) proportionate universalism (universal services with targeted areas for those priority populations at particular disadvantage), and (3) accessible oral health services and better integration of oral and other health services.

Infancy, childhood and adolescence are acknowledged as key life stages in the National Oral Health Plan requiring universal and targeted health promotion input around tooth-friendly diets, brushing and the use tap water, or approved fluoride supplements. The strategic direction of the national plan is aligned with foundation areas, including oral health promotion and improving accessibility of oral health services such as the CDBS. The WA State Oral Health Plan 2016–2020 [18] reflects the direction of the national plan and sets out objectives to support healthy oral health decisions among priority populations. To achieve optimum oral health outcomes in WA and more broadly in Australia, it is critical to understand primary caregivers’ oral health knowledge and behaviours and the associated socioecological determinants.

This review aimed to understand primary caregiver oral health knowledge and behaviours and associated socioeconomic factors within the context of ECC in WA. In order to achieve this, a systematic scoping review was conducted with the following objectives: (1) examine the evidence about current ECC patterns and trends among WA children aged 0–4 years, (2) review the evidence about primary caregivers’ oral health awareness and implementation of positive oral health preventative practices, and (3) the associated socioecological determinants.

## Methods

The five-stage systematic scoping review methodological framework described by Arksey and O’Malley [28] was chosen to guide the systematic scoping review process. The methodology was further checked to ensure it followed the recommendations for a systematic scoping review by Joanna Briggs Institute experts [29]. Given the scarcity of studies and the heterogeneity of existing studies, a systematic scoping review was deemed to be the most appropriate method to achieve the aim of this study. The evidence from this review is drawn from qualitative and quantitative research, as well as publications by state and national health departments and oral health professional bodies.

### Stage 1: Identification of research question

As with any review, the first stage is to consider the research question and the aspects that are of particular interest [28]. For this review, the areas of interest were: (1) current ECC patterns and trends among WA children aged 0–4 years; (2) primary caregivers’ oral health awareness and implementation of positive oral health preventative practices, and; (3) associated socioecological factors. From this, the scoping review also sought to identify the gaps in evidence requiring further research.

### Stage 2: Identifying relevant studies

The main aim of any scoping review is to identify primary studies from a wide range of sources to answer the research question [28]. Our search for literature included using the electronic databases Scopus, PubMed, Medline, CINAHL and PsycINFO and hand searching of article reference lists. Grey literature, identified through university library databases and oral health organisation and government websites, was also considered. Professional websites such as the Australian Dental Association and national databases such as the Australian Institute of Health and Welfare, were also examined.

The articles considered were published between 2010 and 2021 to include evidence published during the period the child dental benefit schedule was introduced and the current Australian and Western Australian Oral Health Plans were in force. All included articles were written in English. The lack of articles specific to WA children meant Australian-wide publications were also considered. Inclusion criteria were rates of ECC for children aged 0–4 years, primary caregiver child oral health awareness and practices of tooth brushing, diet, drinks, dentist attendance, and uptake of the CDBS among eligible families. Publications that delineated this information according to sociological factors were also included for consideration.

Systematic and other forms of literature review were excluded, although articles they reviewed were accessed and considered independently. Boolean logic was used, and search terms included synonyms commonly used in literature (Table 1). Several searches were carried out using the ‘population, interest, outcome’ method (Table 2).

**Table 1:** Search terms

Search term	Synonym and truncation
Oral health	Dental caries, decay, oral care, tooth care, oral hygiene, periodontal disease
Young child	Infant* OR child* OR p*ediatric OR pre-school OR early childhood OR aged 0 to 4 years
Oral health status	Data OR trends OR numbers OR statistics OR monitoring OR records
Caregiver awareness	Health knowledge OR health literacy OR health belief* OR attitude* OR perception
Caregiver behaviour	Health behavio*r OR access OR uptake OR practice OR implementation
Oral health practice	Tooth brushing OR teeth brushing OR infant drinks OR infant diet OR dentist OR Childhood Dental Benefit Scheme
Socio-economic-status	Class OR poverty OR income OR disadvantage

**Table 2:** Searches using population interest outcome method

Search	Population	Interest 1	Interest 2	Outcome	Outcome
One	Oral health	Young child		Oral health status	
Two	Oral health	Young child	Socio-economic status	Oral health status	
Three	Oral health	Young child		Primary caregiver awareness	Primary caregiver oral health practice
Four	Oral health	Young child	Socio-economic status	Primary caregiver awareness	Primary caregiver oral health practice
Five	Oral health	Young child		Primary caregiver behaviour	Primary caregiver oral health practice
Six	Oral health	Young child	Socio-economic status	Primary caregiver behaviour	Primary caregiver oral health practice

This study aimed to explore aspects of child oral health among the general Western Australian population. As such, studies focusing on previously identified high risk groups such as refugee or Aboriginal or Torres Strait Islander backgrounds were excluded.

### Stage 3: Study selection

**Fig. 1:**
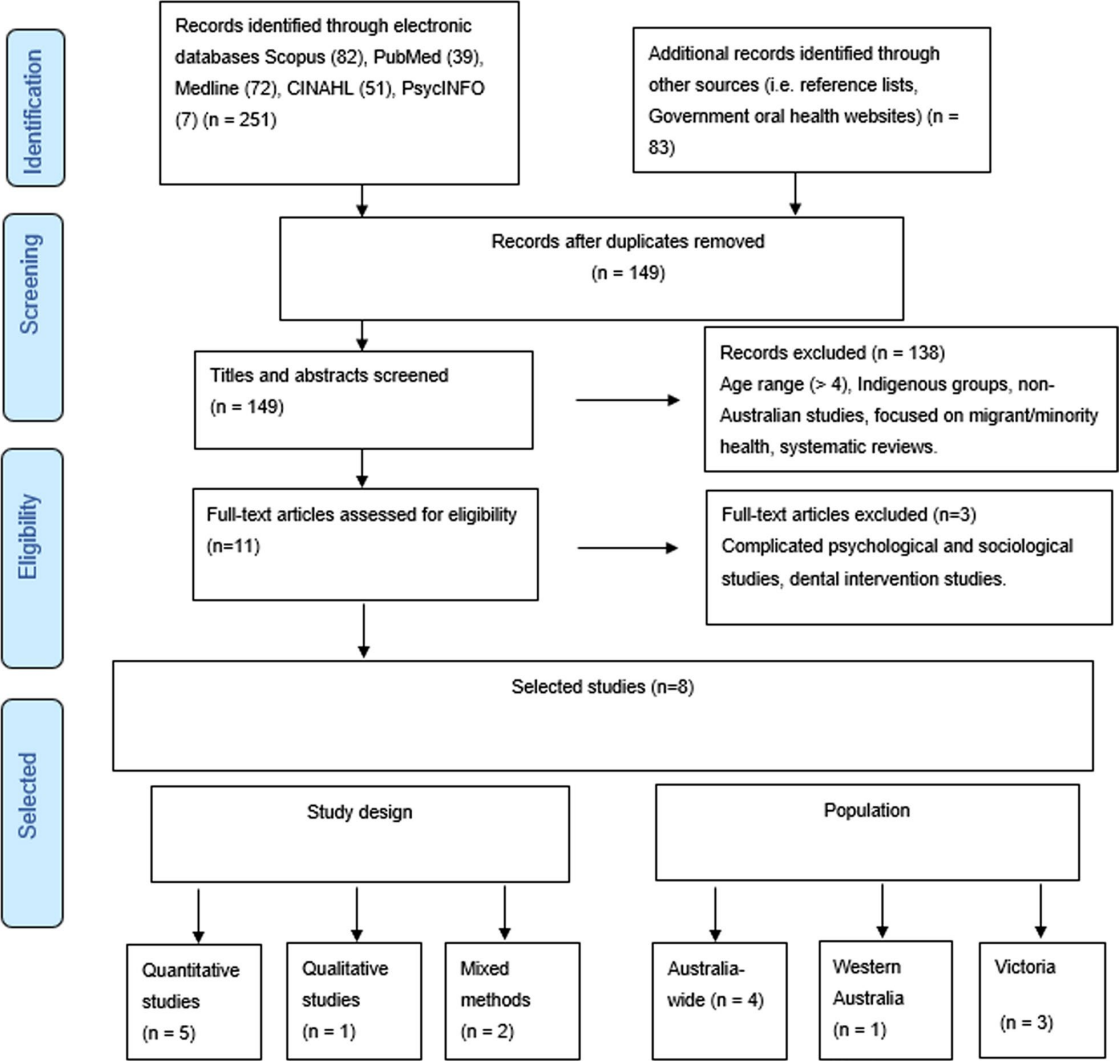
Selection of relevant article process

The process of study selection followed the Preferred Reporting Items for Systematic Reviews and Meta-Analysis (PRISMA) framework [30] (Fig. 1). A total of 251 articles were initially identified through electronic databases Scopus (n = 82), PubMed (39), Medline (72), CINAHL (51), PsycINFO (7) based on the terms defined in Stage 1 [28]. Government and oral health organisation websites and the reference lists of the previously identified studies were checked manually, resulting in 83 further potential articles. Once duplicates were removed, the resultant 149 articles were screened through examination of their titles and abstracts. All these articles were checked by the two lead authors (LA and RW) using the predetermined inclusion and exclusion criteria to achieve consensus. From this exercise, 138 articles were rejected. Three authors (LA, RW and NW) then reviewed the resultant 11 articles and a further 3 were rejected because they were complex sociological or psychological studies or focused on dental treatment.

### Stage 4: Data charting

In this stage of the process, the selected studies were charted and sorted according to key issues and themes, providing a ‘narrative review’ [28] that presents broad information about the study objective, population group, study design/methodology and key findings (Table 3). The eight studies that were included in the final review, reflects the lack of research in this space. Only one was a WA study [31], three were from Victoria [32–34] and the remaining four had an Australian-wide focus [23, 35–37]. Most studies were quantitative in research design, with three analysing different aspects of data from the National Longitudinal Study of Australian Children (LSAC), a longitudinal cohort health and development study of 10,000 children that commenced in 2003 [38]. Most of the findings of interest to our review therefore concerned data collected in 2003 and 2007, when the children in question were aged between 0 and 4 years. Findings were analysed and organised under the themes identified in the literature: ECC trends, primary caregiver awareness and behaviours and socioecological indicators. The second theme was divided into the child’s diet (food and drink, especially sugar-rich), dental hygiene practices (brushing regime and practices that transfer parents’ cariogenic bacteria to the child’s mouth), dental service engagement (frequency of dental visits) and uptake of the CDBS among eligible families.

**Table 3:** Selected articles

Author(s), year published, location	Objective	Population group	Study design/methodology	Key findings/outcomes
Alsharif et al. (2014) Western Australia	To understand the differences in dental insurance cover amongst hospitalised Western Australian children aged 0–15 years, and associated influencing factors	43, 937 WA children aged 0–15 years, hospitalised for an oral-health related condition	Quantitative- data collected from WA Hospital Morbidity Dataset from 1999 to 2009	These finding suggest there are other factors other than the cost of dental care, that prevent parents from accessing oral health treatment for their child. Therefore, health promotion is essential in educating parents of the importance of utilising the scheme
Gussy et al. (2016) Victoria	Identify the ‘natural history’ of dental caries amongst children aged 0–3 years, including risk and protective factors that influence ECC	Birth cohort study that followed 467 mothers and children at age 1, 6, 12, 18, and 36 months	Longitudinal study Quantitative—surveys and oral examinations	Of the 268 children that had a dental assessment at 18 and 36 months- 8% of these children experienced decay, which increased to 23% of children at 36 months The period between age 18–40 months may be a significant period for the development of dental caries, and the following 18 months a period when this may manifest Soft drink (but not fruit juice) consumption was associated with lesion development, likely due to the highly acidic nature of carbonated drinks
Kilpatrick, et al. (2012) Australia-wide	Determine differences between parents reported oral health behaviours for Australian children aged 2–3 and 6–7 years Examine indicators of social disadvantage that may affect oral health (e.g. low SEP) Identify patterns of oral health inequalities between the two age groups	4606 children aged 2–3 years 4464 children aged 6–7 years	Cross-sectional data from the Longitudinal Study of Australian Children (LSAC) Descriptive, mixed-method, two-stage approach Interviews and a questionnaire with the child’s primary caregiver	Children aged 2 to 3 years were less likely than older children (aged 6–7 years) to brush their teeth twice a day (44% compared to 61%) and to have attended a dental health service in the last 12 months (15% compared to 59%) Only 3% of younger children had parent-reported caries, compared to 67% in the older children The most socially disadvantaged were associated with higher odds of caries, infrequent toothbrushing and non-use of dental services
Lucas et al. (2011) Australia-wide	To establish differences in child oral health outcomes and behaviours across Australian states and territories, using the cross-sectional data from the LSAC	As Kilpatrick et al	As Kilpatrick et al	Almost 90% of Western Australian children aged 2–3 years had not accessed dental services, 3 times higher than children in ACT. More than half the WA children (54%) did not brush teeth twice a day and 3% experienced dental caries In the older WA children (aged 6–7 years), access to dental services had increased to 33.3%, brushing teeth twice a day to 30.6%, but dental caries had also increased to 35.5% Interstate differences may be influenced by variations in state-based oral health services and promotion activities
Putri et al. (2020) Australia-wide	To analyse patterns of dental visits under the Child Dental Benefit Schedule (CDBS), the cost of the schedule and utilisation in the first two years of implementation	This study included 678,000 eligible children for CDBS in 2014 and 567,000 eligible children in the following year	Retrospective descriptive analyses- data from Medicare between 2014 and 2015	Eligible children aged 2–4 years utilised CDBS the least WA children had the lowest utilisation per eligible child for preventative services under the CDBS in 2014–2015. However, WA children had higher utilisation of the School Dental Service compared to other states
Rogers et al. (2018) Victoria	To identify the association between rates of oral health hospitalization amongst Victorian Australian children aged 0–4 years and community water fluoridation access, availability of oral health professionals and SES	1297 potentially preventable dental hospitalisations amongst 318,997 children aged 0–4 years living in Victoria, Australia	Cross-sectional-quantitative	Children living in areas with limited access to oral health professionals had 65% higher rates of dental hospitalisation Children from families of low SES had 57% higher rates of dental hospitalisations Children living in areas without water fluoridation were on average 59% higher rates of dental hospitalisations 90% of children were hospitalised because of preventable dental caries
Stormon et al. (2019) Australia-wide	Investigate community-level constructs (e.g. social, physical and community oral health environment) affecting the oral health of children aged 0 to 4 years	10,090 children from the LSAC study	Cross-sectional data from children that participated in the Longitudinal Study of Australian Children (LSAC)—quantitative Used Fisher-Owens et al. (2007) conceptual model to guide the investigation of community level predictors of oral health	Children that were more likely to have caries and dental injury were from low socioeconomic areas, low water fluoridation areas and living in neighbourhoods with poor liveability/facilities Queensland and Western Australia were the states that had the highest odds of children having dental injury at age 4 years
Virgo-Milton et al. (2016) Victoria	Mother’s perceptions, beliefs and behaviours towards child oral health	32 mothers of children aged 4–12 months	Qualitative approach to explore the barriers to promoting children’s oral health—interviews	Themes: meaning of oral health; Causes of poor child oral health (unclear understanding of risk factors); Influences on their child’s oral health (beliefs, parents own experiences, time, child behaviour); Strategies to overcome barriers to poor oral health (toothbrushing, oral health care); Sources of oral health advice (dental professionals, child health nurses, grandparents)

### Stage 5: Collating, summarising and reporting the results

In this stage of the process, the findings from the selected articles are presented. As a scoping review, the findings are discussed as a narrative rather than an assessment of the weight of the evidence [28]. Three themes informed this review: (1) early childhood caries among WA children, (2) primary caregiver oral health knowledge and behaviours, and (3) socioecological factors (socioeconomic and environmental determinants) (all three themes broadened to Australia-wide as necessary).

### Stage 6: Consultation stage

Consultation was undertaken at this stage with JP, Specialist Paediatric Dentist and Senior Lecturer in Clinical and Paediatric Dentistry. As a quality improvement initiative, JP was invited to read the selected articles, provide a final decision on articles contested for inclusion by RW and LA, review drafts and provide comments and feedback.

## Results

### Early childhood caries in Australian and WA children

Although several studies have assessed the oral health of school-aged children, few have done so with pre-school children, with just one study providing data on early childhood caries (ECC) in WA [36]. In this study, 2006 LSAC data revealed ECC among WA children aged 2–3 years in the LSAC cohort was 2.9%, slightly lower than the national average of 3.3%. Only ACT and SA had lower reported rates of ECC at 2.5% and 2.7% respectively. Despite this, WA had the highest proportion (89.4%) of children that had not accessed dental services in the last 12 months. Although this study uses a sample considered to be representative of the Australian child population, the oral health data obtained was primarily based on parental reporting rather than clinical examination. Furthermore, the ECC data for children aged 2 to 3-year, which was gathered during wave one of seven waves of datal collection, was collected in 2004, limiting its relevance to the situation for children of this age in the present time.

In Victoria, Gussy et al. [32] went some way to address the nation-wide gap in ECC data with their cohort study, which followed 467 Victorian children aged 0–36 months (with participants dropping to 269 by the 36-month intervention). This study provides insight into the ‘natural history’ of caries development among very young Australian children, revealing 8% of participants had caries at the age of 18 months, increasing to 23% by age 36 months. While this study offers some evidence, the relatively small sample and high attrition present as limitations.

Publications that present national data, such as Lucas et al. [36] rely on secondary analysis of LSAC data. As with Gussy et al. [32], Stormon et al. [37] and Kilpatrick et al. [35] found ECC increased steeply in children between the age 18–36 months. All three studies associated this increase in rates of ECC with changes in the children’s diets, the introduction of sugar-sweetened beverages (SSB) and the recommendations for increased regular tooth brushing during this developmental stage not being followed. All three publications concluded that the prime time for oral health promotion and intervention strategies for young children was between the age of 18 and 36 months. The need for early intervention to halt or slow ECC progression is further supported by additional LSAC data stating that rates of ECC among 6 to 7-year-old children was tenfold that of 2 to 3-year-old children [35]. Again, the dates of the national data analysed in these studies limit their relevance to the contemporary child oral health situation.

### Primary caregiver oral health knowledge and behaviours

The LSAC also provided information on primary caregiver oral health knowledge and behaviours. Kilpatrick and colleagues [35] revealed that Australia-wide, among the primary caregivers of children aged 2–3 years, just 44.4% reported brushing their child’s teeth twice a day (increasing to 67% for children aged 6 to 7 years). Only 15.2% of primary care givers had taken their child (aged 2–3 years) to a dental health professional in the preceding 12 months, increasing to 59.4% for children aged 6–7 years [35]. A state-by-state analysis of tooth brushing behaviour by Lucas et al. [36] found WA had the highest rate for correct tooth brushing regimes for children aged 6–7 years, however, more than half of these children experienced suboptimal toothbrushing at the earlier age of 2–3 years.

Analysis of the LSAC by Stormon et al. [37] found Australian mothers’ knowledge on dietary practices was inconsistent: most knew about the cariogenic effect of SSB but few knew about the potential harm associated with night time feeds or the transfer of caries inducing bacteria to their children through poor dental hygiene practices such as kissing children on the lips and sharing feeding utensils.

In Victoria, Virgo-Milton et al. [34] interviewed the mothers (n = 32) of young children (aged 4–12 months) about good oral health, reporting that those with lower oral health knowledge may be more likely to delay seeking treatment for their child. Conversely, those who had experienced negative dental experiences during their own childhood, were reported as placing greater impetuous on providing good oral health care for their own children. Australia-wide 2014–2015 Medicare data on children eligible for the CDBS revealed children aged 2–4 years were utilising the schedule the least and most primary caregivers who did access the schedule, did so for their children aged 5–17 years [39]. Furthermore, specific to WA, although 28% of children were eligible for the CDBS only 4% accessed the scheme in 2014, increasing to 6% in 2015.

### Socioecological determinants

Kilpatrick et al. [35] reported that for the Australian children in the LSAC study “marked social disparities in oral health appear as early as 2 years of age and remain evident in school-age children” (p. 38). Social indicators for poorer oral health included rural location, English as an additional language and parental income, employment and housing. The authors recommended targeted oral health promotion interventions as early as possible in the child’s life to mitigate these disparities.

In WA, records of oral health hospital admissions for children aged 0–4 years showed 82.7% from the lowest socioeconomic status quintile had no dental insurance, compared to 32% from the highest quintile [31]. Rural location was also associated with higher hospitalisations and lower dental insurance for children under 5 years [31]. The rate of dental insurance for children aged 0–4 years in WA decreased by 22% between 2000 and 2009. Lucas et al. [36] argued that existing child oral health disparities between Australian state or territory of residence, when corrected for individual sociodemographic determinants, were the result of differences in parental understanding of good oral health practices, accessibility of public dental services, the different efforts made between states and territories to promote and support oral health and insufficient engagement with oral health professionals.

Fluoride levels in tap water are a further factor requiring acknowledgement in a discussion on social and environmental determinants of child oral health, as the link between fluoride exposure and dental caries is well established [40]. Both Rogers et al. [10] and Stormon et al. [37] reported that children aged 0–4 years children living in areas without water fluoridation had significantly higher rates of dental caries and preventable hospital admissions for oral health issues.

## Discussion

### Implications for future oral health promotion activities

The findings are interpreted against the systematic scoping review purpose, namely to, (1) examine the evidence about current ECC patterns and trends among WA children aged 0–4 years (2) review the evidence about primary caregivers’ oral health awareness and implementation of positive oral health preventative practices, and (3) the associated socioecological determinants (with Australian-wide data included where relevant).

### Gaps in existing data

The WA State Oral Health Plan argues research and evaluation are key strategies to addressing poor oral health: “A structured and coordinated research and evaluation program is required to inform the development of appropriate, effective and sustainable oral health services” [18], p. 2]. Nevertheless, the findings of this systematic scoping review reveal significant gaps in the data required to achieve this goal for WA’s young children. Data regarding early childhood caries in WA children are absent. The only figures available come from the LSAC, which reported 2.9% of WA children aged 2–3 years had caries [36]. Given these figures are over 15 years old, it is impossible to use them to inform current WA patterns and trends in ECC, and as such it is impossible to develop, implement and evaluate effective and meaningful oral health promotion programs and strategies. For the same reasons, the Australian-wide data arising from the LSAC is also limited in its usefulness. Other Australian-wide data that indicate a sharp increase in ECC incidence in children between the ages of 18 months and 36 months suggests oral health promotion strategies are required as early as possible in the child’s life.

### The oral health knowledge and behaviours of primary caregivers in Australia

It is evident that Australia-wide, many primary caregivers have insufficient levels of oral knowledge to effectively achieve optimum oral health outcomes for their very young children. Primary caregiver’s behaviours around their children’s toothbrushing is suboptimal [25, 35, 36]. Routine dental attendance for prevention and screening is also inadequate, with vital opportunities to improve primary caregiver oral health knowledge and practices with their young children and curtail the advancement of early childhood caries being lost [35, 36]. These findings concur with the National Child Oral Health Study 2012–2014 [41]. While the focus of this report focus was children aged 5–14 years, it revealed that just 54% of children had visited a dental health professional before the age of 5 years.

A particular primary caregiver oral health behaviour seemingly requiring attention is the practice of sharing utensils and food with children, which exposes them to cariogenic bacteria. Although several studies have attempted to reduce vertical transmission, the colonisation of the oral cavity by cariogenic bacteria is likely inevitable with interventions aiming to prevent vertical transmission, making little impact in reducing levels of ECC [42, 43]. Nevertheless, advice and guidance around about bedtime feeding practices detrimental to oral health is needed as part of a holistic oral health plan for young children [34, 37].

Although these Australia-wide studies reported primary caregivers had a better knowledge about the effects of SSB on their children’s teeth [37], they also demonstrated that the diets of South Australian children aged 0– years contained free sugars in excess of WHO recommendations [26] and contrary to the Australian Dietary Guidelines recommendation that SSB should not be provided to children younger than 12 months [44]. This finding has been echoed in a New South Wales study on child obesity, which found 42.7% of its participant mothers reported they introduced SSB in their child’s diet before the age of 12 months [45]. Nutrition plays a significant role as the dietary experiences of a child in the first 1000 days of life have a profound impact on eating behaviours and food choices throughout life [46], thus healthy food promotion strategies need to incorporate messages about oral health appropriate foods and drinks [47].

An associated issue that requires consideration in any oral health strategy aimed at young children today is the recent exponential growth of the Australian baby food industry [48]. The market is increasingly flooded with sugar rich infant and toddler products, with the majority of ‘pouch foods’ increasingly available in the supermarket being predominantly sweet [49]. Although these foods were not discussed in the reviewed articles, perhaps because of their recent establishment in the baby and toddler food market, their sugar content is concerning given the well-established links to dental caries44. The smooth texture of these foods also warrants attention, as they can encourage persistent and prolonged ‘sucking’ feeding in which the teeth are bathed for longer periods to this sugar-rich food. One way to reduce the high sugar content in very young children’s diets is to provide support and education to primary caregivers around these convenience foods.

### Australia-wide evidence of child oral health inequity

This systematic scoping review identified several socioecological factors that contribute to the inequitable incidence of ECC among very young Australian children, including socioeconomic status and geographic location.

The importance of accessible preventative oral health services in reducing ECC is evident from Government data that reveal children living in areas with limited access to oral health professionals have a 65% higher rate of dental hospitalisation [27]. Several socioecological factors can influence primary caregiver engagement with these services. Family financial status is a strong predictor of dental service accessibility, with the high individual responsibility for the cost of dental treatment (57%) is considerably greater than other health services (11%) [27]. This reduces dental service accessibility for children in low-income families and contributes to the inequitable oral health outcomes across the social divide [31].

A concerning finding specific to LSES families in WA is the lack of uptake of the CDBS with only 4–6% of eligible children accessing the scheme in 2015 [23]. This appears puzzling considering primary-care givers in LSES families are more likely to suffer oral health problems themselves and the finding that these individuals tended to place greater impetus on their own child’ oral health [34]. Again, socioecological factors come into play here; a lack of available dental health professionals in LSES communities, especially rural areas, for example, reduces primary caregiver awareness of the schedule and limits access to local dental services in general [33]. The reasons for the lack of uptake of the CDBS may be due to negative parent experiences of dental treatment as a child [50] and parents fear of stigmatisation associated with a perceived ‘dental neglect’ of their children [51].

This review also revealed that Australian communities without water fluoridation experience significantly higher rates of dental caries and other oral health associated hospital admissions among young children [33, 37]. Inadequate tap water fluoridation and the increasing reliance on bottled water among the Australian population [52], may have important implications for child oral health. There is evidence to suggest parents believe bottled water is a healthier option for their children, due to a combination of a general ‘mistrust’ of tap water and persuasive bottled water marketing campaigns [53]. A combination of public health policy action plus primary caregiver oral health promotion activities that target the insufficient exposure of young children to fluoride is required.

### The need for WA evidence

While the primary caregiver oral health knowledge and behaviour evidence provided by this systematic scoping review is enlightening, most is not WA focused. The early childhood caries statistics are dated, inconsistently recorded and tend to be Australia-wide, parent-reported or from ‘other’ Australian states. Differences exist between states and territories such as population access to public dental health services and efforts made to promote and support community and family oral health [36]. Other important differences include levels of family poverty, rural and remote factors, tap water fluoride levels and the availability. This heterogeneity of socioecological factors and oral health services mean relying on this evidence in planning WA specific interventions is problematic.

Although not the intended focus of the scoping review, the findings reveal a broader lack of consistent, reliable and contemporary oral health evidence across Australia.

## Recommendations

It is evident that WA specific research is required to inform universal and targeted intervention strategies that meet the oral health needs of young WA children and their families. Primary caregiver oral health knowledge and behaviours needs to be ascertained, ongoing reliable ECC data need to be gathered and the socioecological factors influencing the two identified.

To achieve the goals and objectives of the WA State Oral Health Plan, policy change is also needed. It can be argued that, to achieve reliable dental caries records for children aged 0–4 years, greater access to free public dental services is required, facilitating regular family access to preventative dental services and treatment. The adoption of the WHO recommendation that ECC risk assessments should occur by the time a child is 12 months old and re-evaluated regularly would provide an opportunity for the routine collection of ECC data, which in turn could direct targeted oral health promotion strategies. At the time of writing this systematic scoping review, the WA Labour Party manifesto has promised to introduce free dental check-ups for all children aged 6 months to 5 years [54]. If this eventuates, this may go some way to improve dental service engagement across all social groups and enable more reliable ECC data collection in very young children.

More broadly, the lack of Australia-wide contemporary evidence revealed by this scoping review across ECC data and primary care-giver knowledge and behaviours, coupled with the evidence of social determinant related oral health inequity for young children highlights the need for nation-wide research if the goals of the National Oral Health Plan are to be met. This review also argues that national Government action is required in the promotion of the Child Dental Benefit Schedule and the protective benefits conferred by adequate fluoride exposure whether it be through water fluoridation or use of fluoridated toothpaste for young children.

## Conclusions

Oral health related childhood preventable hospitalisations and dental general anaesthetic procedures are increasing among Australian children. The WA State Government has indicated a commitment to reducing this oral health burden, proposing the development of a universal and, where indicated, targeted health promotion approach with children and their families, with a particular focus on the range of socioecological determinants influencing oral health outcomes for the state’s children. This scoping review has demonstrated the lack of ECC data and evidence of primary care-giver oral health knowledge and behaviours in Western-Australia.

This situation means WA health promotion professionals are limited in their ability to inform and evaluate future oral health initiatives. Research with WA primary caregivers is urgently required to inform practice and highlight necessary structural and policy factors that currently disadvantage the oral health outcomes for some WA children and to realise the WA State Oral Health Plan.

## Data Availability

All data generated or analyzed during this study are included in this published article. Details of all studies analysed in this scoping review are included in Table 3 in this article.

## Abbreviations

AIHW: Australian Institute of Health and Welfare
CDBS: Child Dental Benefit Schedule
ECC: Early childhood caries
LSAC: Longitudinal Study of Australian Children
SSB: Sugar sweetened beverages
WA: Western Australia
